# Evolving BioAssay Ontology (BAO): modularization, integration and applications

**DOI:** 10.1186/2041-1480-5-S1-S5

**Published:** 2014-06-03

**Authors:** Saminda Abeyruwan, Uma D Vempati, Hande Küçük-McGinty, Ubbo Visser, Amar Koleti, Ahsan Mir, Kunie Sakurai, Caty Chung, Joshua A Bittker, Paul A Clemons, Steve Brudz, Anosha Siripala, Arturo J Morales, Martin Romacker, David Twomey, Svetlana Bureeva, Vance Lemmon, Stephan C Schürer

**Affiliations:** 1 Department of Computer Science, University of Miami, 1365 Memorial Drive, 33146 Coral Gables, FL, USA; 2 Center for Computational Science, University of Miami, 1320 S. Dixie Highway, Gables One Tower, 33146 Coral Gables, FL, USA; 3 The Miami Project to Cure Paralysis, 1095 NW 14th Terrace, 33136 Miami, FL, USA; 4 Department of Molecular and Cellular Pharmacology, University of Miami School of Medicine, 1120 NW 14th Street, CRB 650 (M-857), 33136 Miami, FL, USA; 5 7 Cambridge Center, Cambridge, MA 02142, MA, USA; 6 Novartis Institutes for BioMedical Research, 250 Massachusetts Avenue, 02139 Cambridge, MA, USA; 7 Thomson Reuters, 5901 Priestly Drive, Suite 200, 92008 Carlsbad, CA, USA

## Abstract

The lack of established standards to describe and annotate biological assays and screening outcomes in the domain of drug and chemical probe discovery is a severe limitation to utilize public and proprietary drug screening data to their maximum potential. We have created the BioAssay Ontology (BAO) project (http://bioassayontology.org) to develop common reference metadata terms and definitions required for describing relevant information of low-and high-throughput drug and probe screening assays and results. The main objectives of BAO are to enable effective integration, aggregation, retrieval, and analyses of drug screening data. Since we first released BAO on the BioPortal in 2010 we have considerably expanded and enhanced BAO and we have applied the ontology in several internal and external collaborative projects, for example the BioAssay Research Database (BARD). We describe the evolution of BAO with a design that enables modeling complex assays including profile and panel assays such as those in the Library of Integrated Network-based Cellular Signatures (LINCS). One of the critical questions in evolving BAO is the following: how can we provide a way to efficiently reuse and share among various research projects specific parts of our ontologies without violating the integrity of the ontology and without creating redundancies. This paper provides a comprehensive answer to this question with a description of a methodology for ontology modularization using a layered architecture. Our modularization approach defines several distinct BAO components and separates internal from external modules and domain-level from structural components. This approach facilitates the generation/extraction of derived ontologies (or perspectives) that can suit particular use cases or software applications. We describe the evolution of BAO related to its formal structures, engineering approaches, and content to enable modeling of complex assays and integration with other ontologies and datasets.

## Background

### Introduction to BAO and the domain

The development of novel small molecule therapeutics (drugs) typically begins with the identification of suitable compounds with desirable biological activity in simple model systems such as a purified protein that is a validated disease target or a cell related to a disease or disease state. Target-based and cell-based phenotypic high-throughput screening (HTS) are among the most important approaches to identify new hits and leads from large compound libraries [1,2]. Innovations in assay design and technological advances in detection and throughput have dramatically increased the size and diversity of HTS datasets generated in pharmaceutical companies and in public research projects. Examples of NIH-funded large-scale screening programs in which we have been participating include the Molecular Libraries Program (MLP) [3] and the Library of Integrated Network-based Cellular Signatures (LINCS) program [4]. In the MLP, a large library (up to 430, 000 compounds) has been screened in over 600 probe projects to develop novel tool and drug compounds. This data is deposited in PubChem [5] and is also being curated and made available for structured analysis in the BioAssay Research Database (BARD) [6]. The LINCS project, in contrast to traditional screening, generates extensive signatures of cellular responses consisting of thousands of results for any perturbation (such as small molecule drugs) to enable the development of better system-level disease models. Examples of LINCS screening results and assays include Landmark gene expression signatures (L1000), Kinome-wide binding affinities (KINOMEscan), phenotypic profiling across 1,000 cell lines, and many others, covering "omics" and HTS data. LINCS results are currently available via participating centers and can be queried and explored via the LINCS Information FramEwork (LIFE) developed by our group [7]. Several other publicly accessible resources of screening data exist, for example ChEMBL, a database that contains structure-activity relationship (SAR) data curated from the medicinal chemistry literature [8], the Psychoactive Drug Screening Program (PDSP), which generates data from screening novel psychoactive compounds for pharmacological activity [9], or Collaborative Drug Discovery (CDD), a private company enabling drug discovery research collaborations [10].

Despite being publicly available, current data repositories suffer from structural, syntactic, and semantic inconsistencies, complicating data integration, interpretation and analysis. As one of the largest and first repositories of public drug screening data, PubChem, has been essential to illustrate the need for clear metadata standards to describe drug and chemical probe discovery assays and screening results [11]. To address these prevailing issues; we have previously developed the first version of the BioAssay Ontology (BAO) [12]. This first version was developed iteratively based on domain expertise and available assay data, primarily from the MLP, which we annotated using evolving versions of BAO. Since the first release of BAO, we have engaged with several more groups in public research projects and in pharmaceutical companies and the biomedical ontology community. We aligned the organization of BAO with existing efforts as much as possible, most importantly at the Novartis Institutes of BioMedical Research, and we have significantly extended the terminology and axioms in BAO to cover a broader range of assays and related concepts. One of our objectives in redesigning BAO was to introduce an upper-level ontology to facilitate alignment and integration with other biomedical domain ontologies and to provide a more formal ontology development framework. However, a critical requirement was to maintain a "native" organization of BAO that is meaningful to end users and which enables straight-forward incorporation into software systems, such as our previously developed BAOSearch application [13]. This led us to a formal, structural, and functional modularization of BAO, which we describe here. We also provide a general solution to defining profile and panel-type assays in which many results are generated in parallel, such as those in the LINCS project. Meanwhile, BAO has been applied in several new projects, most importantly BARD, which also contributed to extending and improving BAO further.

Semantic Web technologies have become increasingly popular to integrate biomedical research information; a prominent example is the Bio2RDF project [14]. In addition to "open-world" integration of diverse omics and high-throughput drug screening data, Semantic Web technologies provide capabilities for inference reasoning with many potential benefits over traditional systems [15]. Only very recently however, have large public drug screening datasets been made available as Resource Description Framework (RDF) format. One such resource is ChEMBL, whose RDF model leverages BAO to describe the results [16]. A large initiative to develop an integrative solution to diverse drug discovery data is the Open Pharmacological Concepts Triple Store (Open PHACTS) consortium [15]. Because of increasing adaptation of Semantic Web technologies in drug discovery data management, it was critical to develop BAO as a formal Description Logic (DL) ontology implemented in Web Ontology Language (OWL). We show modeling examples illustrating BAO semantic inference capabilities to identify mechanistically related assays in absence of such explicit annotation.

### Description logic

Description logic (DL) contains a set of decidable constructs from the first-order predicate logic, and it is the corner stone for the development of OWL DL ontologies in knowledge representation [17]. The computational complexity of a given DL depends on the constructs that are being used, and they are traditionally represented with different complexity classes. Attribute Language with Complement (ALC) provides the preliminary DL constructs with classes, roles, and individuals. The formal syntax of ALC is defined as follows (as a convention, we indicate conceptualization by capital letters (e.g., *C, D*) or sans serif letters (e.g., Thing, bioassay), sets by bold face letters (e.g., **C**, **I**), and functions by lower case letters (e.g., *f***_C_**,*f***_I_**)). Let *A *be a named atomic class, and, without loss of generality, let *R *be an abstract role. The class expressions (concepts or concept expressions) *C, D *are recursively constructed by: *C, D *← *A *| ⊤ | ┴ | ￢*C *| *C *⊓ *D *| *C *⊔ *D *| ∀*R.C *| ∃*R.C*, where, ⊤ is the top concept, ┴ is the bottom concept, the symbols for conjunction, disjunction, and negation are given by ⊓, ⊔, and ￢ respectively, and ∀ and ∃ represent the universal and existential quantifier. ALC DL knowledge bases consist of two groups: (1) TBox provides statements about the terminological knowledge; and (2) ABox provides the statements about the assertional knowledge about individuals. These statements are also known as axioms in description logic. For class expressions *C *and *D*, the TBox statements are of the form *C *≡ *D *or *C *⊑ *D*, where ≡ denotes the equivalences among classes and ⊑ constructs the subsumption or general class inclusion (GCI) axioms. On the other hand ABox consists of axioms of the form *C*(*a*) and *R*(*a, b*), where *R *is a role, and, *a, b *are individuals.

ALC DL has been extended to SROIQ(D) DL with the following syntactic constructs: {*a*} | ∃*R*.Self | ≤ n*R.C *| ≥ n*S.C*, where, {*a*} represents nominals, ∃*R*.Self relates an individual to itself, and n ∈ ℤ^+ ^with ≤ n*R.C *and ≥ n*S.C *provide the qualified cardinality restrictions. SROIQ(D) DL introduces an RBox with general role inclusion axioms of the form *R*_1 _. . . *R*_2 _⊑ *R*, which provides the meaning that concatenation of *R*_1_*, . . . , R*_2 _is a subrole of *R*. In addition, there exists constructs to represent transitive, symmetric, asymmetric, reflexive, irreflexive, functional, inverse functional, and disjoint roles and concepts. It is to be noted that roles can either be abstract or concrete.

The interpretation of DL is given by the direct model-theoretic semantics. The classes, roles, and individuals are given symbols from mutually disjoint sets of **C**, **R**, and **I **respectively. There exists another set called the domain of interpretation, ∆, which contains entities for resources, individuals, or single objects. Using the domain of interpretation, the individuals, classes, and roles are interpreted by functions *f***_I _**: **I **↦∆, *f***_C _**: **C **↦ 2^∆^, and *f***_R _**: ↦ 2^∆*×*∆ ^respectively. The complex classes and role expressions are interpreted by an extended interpretation function, $.^{I}$ , such that the interpretation faithfully capturers the structure of the knowledge base. If a model exists, then the knowledge base is satisfiable, and the implicit knowledge (logical consequence) is entailed though an inference procedure. DL logic uses efficient tableau algorithms to infer subsumption, class equivalence, class disjointness, global consistency, class consistency, instance checking, and instance retrieval.

DL provides an appropriate trade-off between expressivity and scalability in practice. The complexity of DL is dominated by the data complexity, which is NP-hard for SROIQ(D) DL ABox and N2ExpTime-complete for the combined TBox, RBox, and ABox. Modern SROIQ(D) DL reasoners such as the (1) tableau-based FaCT++ [18] and Pellet [19] reasoners; and the (2) hyper-tableau HermiT [20] reasoner, use intelligent heuristics and optimization methods to perform inferencing as efficiently as possible. The reader is referred to [21,22] for a comprehensive discussion on SROIQ(D) DL syntax, semantics, deduction procedures, and model construction.

## Results and discussion

### BAO 2.0 native organization and main components

The new BAO 2.0 formally describes perturbation bioassays in the domain of drug and probe discovery, such as small molecule HTS assays and screening results for the purpose of categorizing the assays and outcomes by concepts that relate to the screening model system (format), assay method, the biology interrogated in the assay (such as a protein target or biological process), the detection method (how does the assay work), and types of results (endpoints). BAO 2.0 is organized into several major sections, which include multiple levels of subcategories of subsumption class hierarchies. A number of specific object property relationships were created to connect the classes and develop a knowledge representation.

**Figure 1:**
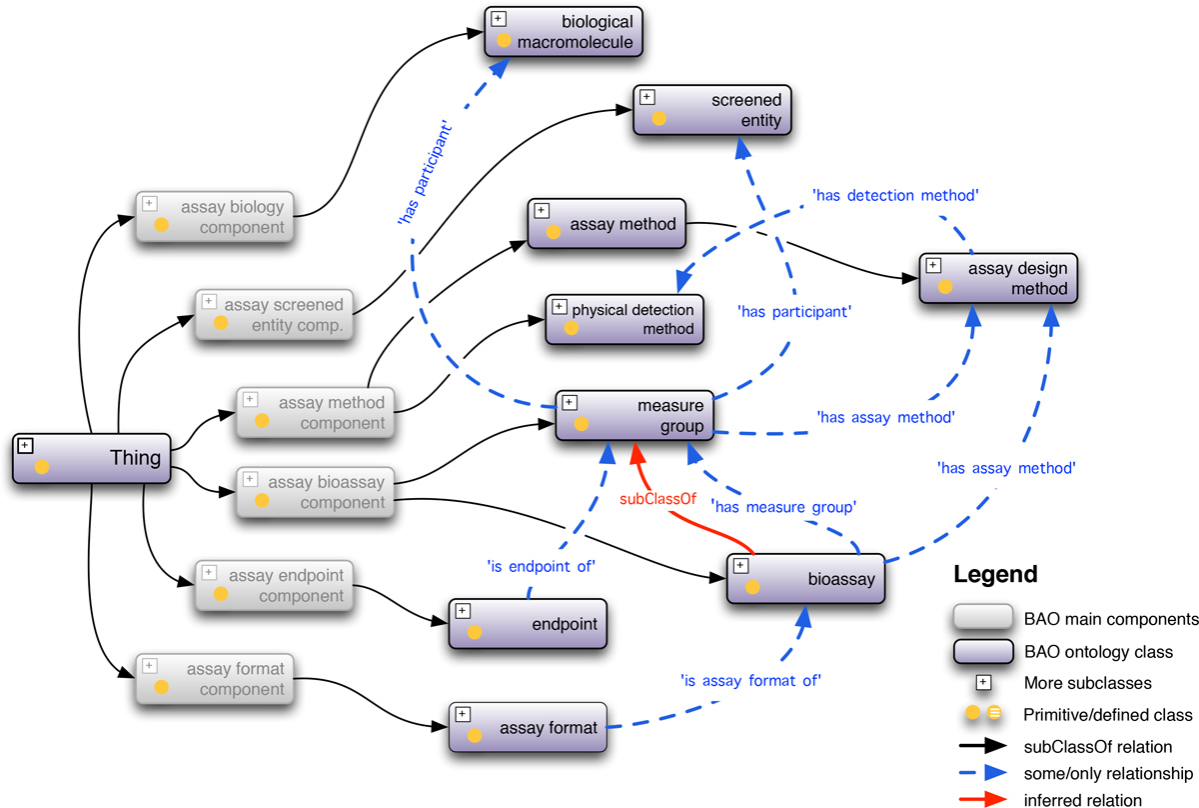
**BAO 2.0 main classes with some relationships between them**. Six main components (shaded classes) are used to formally describe bioassays by terms related to bioassay, biology, screened entity, assay method, format, and endpoint. The most important classes and their relations as shown including bioassay, measure group, biological macromolecule, screened entity, assay method (specifically assay design method and physical detection method), assay format, and endpoint. There exists complex interactions among these entities. OWL DL 2 (*SROIQ*(*D*)), the decidable subset of the first-order-predicate logic provides the interpretations, models, and logical consequences.

The main categories in BAO 2.0, titled components, include bioassay, assay biology, assay method, assay format, assay endpoint, assay screened entity (Figure 1). Each of these component classes includes the subsumption trees of terms corresponding to the category and additional trees of related terms to describe each of the main components properly and formally. In BAO 2.0, we incorporated a slightly different pattern from BAO 1.6, since we were interested in making BAO 2.0 compatible with the existing upper-level and other domain-level ontologies. The BAO 2.0 categories also lend BAO to its native structures that is most useful to users, for example to annotate assays or to implement a user interface in a software application. We describe briefly the main class hierarchies of BAO 2.0 corresponding to the above components (Figure 1):

• BAO assay bioassay component includes the bioassay subsumption tree, and several other classes to describe assays, including assay kit, bioassay type, and bioassay specification, which contains terminology trees to describe various details about a bioassay and its context. The class hierarchy bioassay includes the list of the bioassays and their formal description, e.g., cell cycle assay, enzyme activity assay. Bioassays are organized roughly by their application (what the assay is used for). The class hierarchy assay kit includes the reagents and their cocktails that are commercially available to perform the different chemical reactions that encompass an assay (i.e., out of the box, ready to run assays). The information in bioassay specification is similar to BAO 1.6.

• BAO assay format component includes the assay format subsumption tree to describe the biological model system; a conceptualization of assays based on the biological and/or chemical features of the experimental system.

• BAO assay method component includes terminologies to describe how the assay is performed, most importantly assay method and physical detection method. It also includes computational method, instrument, and relevant other material entity "assay ingredients". The class hierarchy assay method includes assay design method and assay supporting method; assay design method describes how a biological perturbation of the model system is translated into a detectable signal. The class hierarchy computational method contains various methods that are based on the application of information technology to chemistry and biology. The physical detection method hierarchy includes the method (technology) used to detect the signal that corresponds to the perturbagen in the assay environment and enabled by the assay design method. Class instrument consists of instruments used for detection/readout from an assay and their components, e.g., FLIPR, ViewLux plate reader, PHERAstar, etc; software lists the types of software that are used in the various instruments, e.g., image analysis software, which is a component of the high content screening (HCS) platforms.

• BAO assay biology component includes various class hierarchies to describe the biology of the assay including biological process, biological macromolecule, cell line cell, cellular component, cell phenotype, anatomical entity, disease, function, organism. Many of these are mapped to external sources (*vide infra*). To describe the biology of a simple binding assay for example, a biological macromolecule protein would have the biological role target. Many other role classes exist (*vide infra*). The class function includes the physiological function of biological macromolecules, e.g., protein binding, kinase activity. This module was imported from the Gene Ontology (GO). The class cellular phenotype encompasses both the molecular characteristics of a cell and the (morphological) shape and structure of a cell and its parts.

• BAO assay screened entity component includes screened entity, which is the chemical or biological entity that is tested/screened in the assay. The screened entity typically modulates the function of the (known or unknown) biological macromolecule with the role of a target. The most important screened entity for BAO is the class small molecule, that contains compounds that are tested in the process of developing chemical probes and drugs, which is the primary domain of BAO.

• BAO assay endpoint component includes subsumption trees to describe the assay result or endpoint and other required information to quantitatively or qualitatively express the biological perturbation measured in a bioassay, such as units of measurement (imported from UO), and other details to interpret the results in the context of the assay methodology and the biology, such as as the mode of action of the perturbagen that the endpoint characterizes, or the signal direction and endpoint action correlation of the assay. More details about the class endpoint are described below.

• Additional classes that were not assigned to any one of the main BAO components are organization, people, role, and quality: Organization includes, for example manufactures of assay kits, instruments, etc., or screening center where assays are performed. People include the individuals who are involved in performing scientific research, such as assay development, compound screening, chemical synthesis, etc. Role describes the action that an entity performs in a given context; an entity can have more than one role, e.g., target, perturbagen. BAO 2.0 has imported roles from the Chemical Entities of Biological Interest (ChEBI) ontology and we have added some missing classes. Quality lists the characteristics that inhere in an entity of biological origin, namely, organism, cell, and molecule or a physical entity, e.g., intensity, optical quality. Most of the terms in this class were imported from the Phenotypic Quality Ontology (PATO); missing ones were added to BAO.

• BAO properties include both the object and data types that are required to create relationships among the different concepts in BAO 2.0. These properties were either imported from the Relationship Ontology (RO), where available or created in BAO 2.0.

### Upper level ontology structure and aligning external ontologies

**Figure 2:**
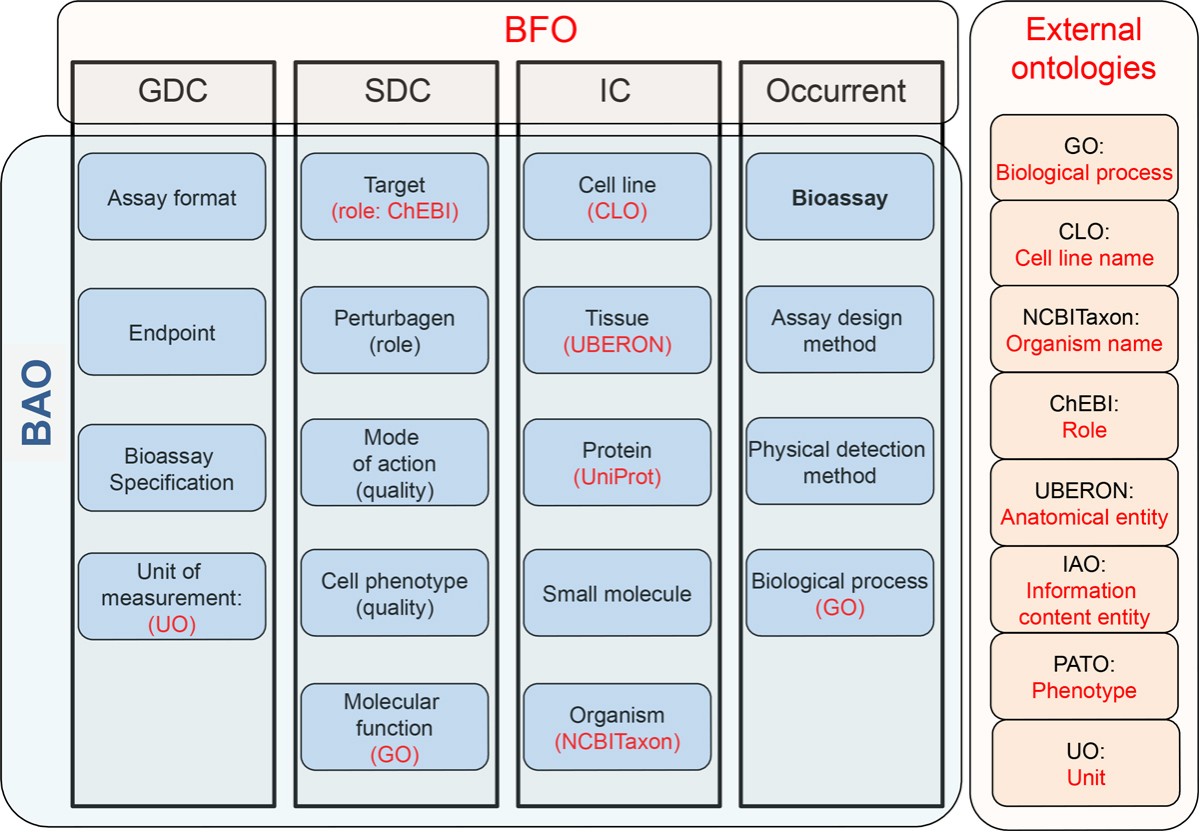
**BAO 2.0 makes use of BFO as the upper-level ontology and incorporates several external ontology modules**. BAO classes were mapped under appropriate BFO concepts. The BFO framework also facilitates alignment to external ontology modules. Blue boxes are examples of BAO classes categorized by BFO (black rectangles). External ontologies used in BAO are shown as red boxes and labels.

Since, there are several advantages of using upper level ontologies (ULOs), BAO 2.0 makes use of the Basic Formal Ontology (BFO) and OBO Relations Ontology (OBO-RO) as its upper level ontologies. We have used the current release of BFO ontology (http://purl.obolibrary.org/obo/bfo.owl), which is also tightly coupled with OBO-RO ontology (http://purl.obolibrary.org/obo/ro.owl). Figure 2 shows the main categories of BFO and examples of corresponding BAO 2.0 classes. BFO conceptualization abstractly represents objects, entities, and relations in our domain of discourse, and it is substantially used in biomedical ontologies compared to other OWL version of ULOs such as SUMO (http://www.ontologyportal.org/SUMO.owl) or DOLCE (http://www.loa.istc.cnr.it/ontologies/DLP 397.owl). The advantage of using an ULO is that it allows integration of existing domain ontologies, by grounding them on a formally rigid ontological framework [23,24]. We make available a development instance of the BAO BFO version. Figure 2 also illustrates external ontologies, components of which we currently use in BAO (see Methods). Their alignment with BAO is facilitated in part by the BFO structure [25,26]. One important mid level ontology is the Ontology for Biomedical Investigations (OBI) [27]. We have previously outlined the different focus of BAO vs. OBI [12]. However, this is not to say that they are incompatible; alignment is one of the future tasks required to evolve BAO further. We have created a version of BAO 2.0 that contains BFO and OBO-RO as ULOs (bao_complete_bfo_dev, which is a development version) and another one without them (bao_complete, released). Bao_complete_bfo_dev simplifies alignments to external ontologies and is targeted to the ontology development community while bao_complete is targeted to the drug and probe screening community and developers of software applications (such as our BAOSearch application). We emphases the fact that the BAO-to-BFO alignment is based on our knowledge and understanding of BFO and OBO-RO structures, and BAO mechanisms. The alignment is an ongoing process, and we have a community wide bug reporting system to uses to provide feedback to provide semantically better alignments. bao_complete_bfo_dev and bao_complete are targeted towards different users groups, the latter is more amenable to perform on large-scale analysis of the chemical biology data without the additional constraints imposed by BFO and OBO-RO.

### BAO 2.0 modular architecture and implementation

**Figure 3:**
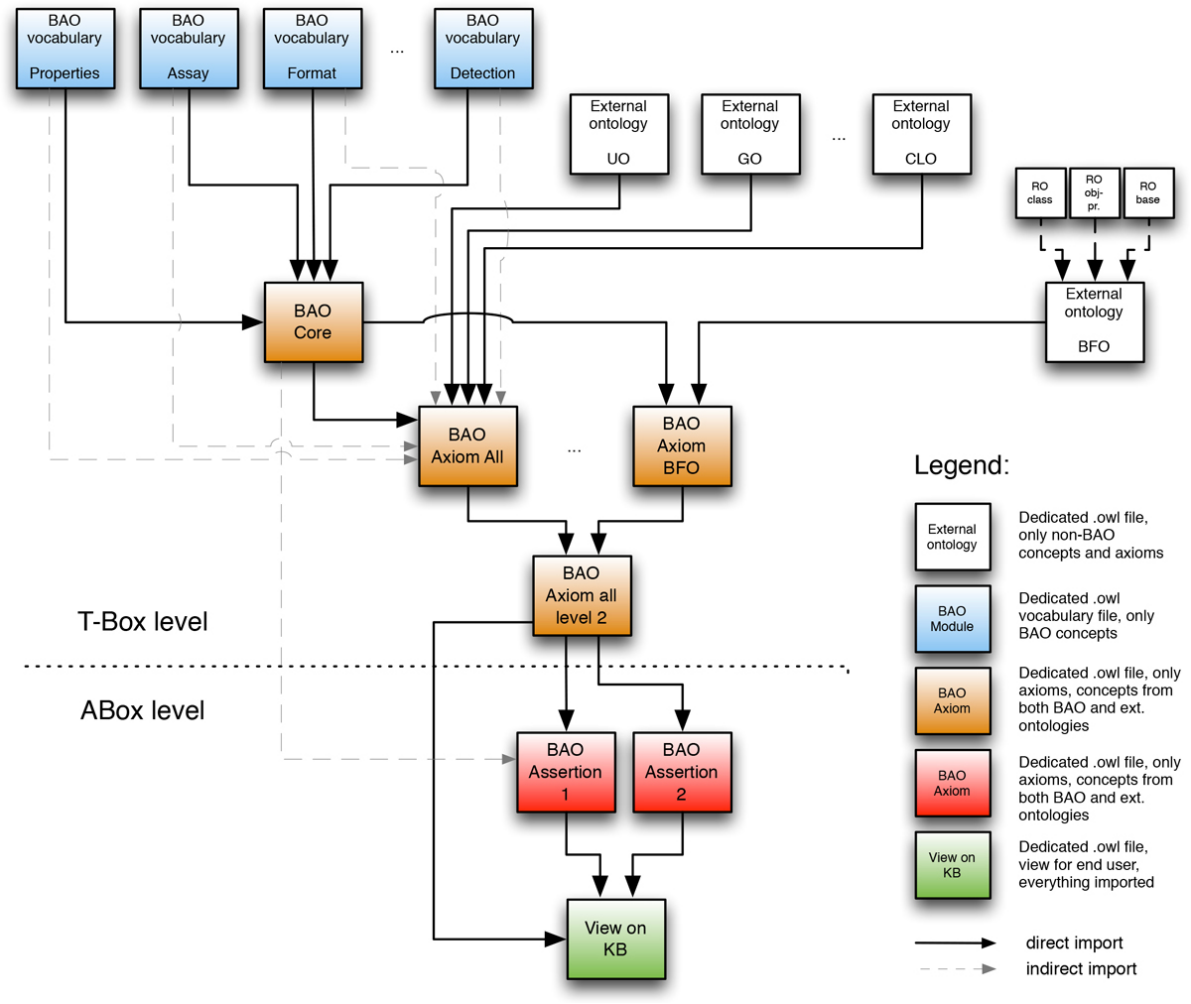
**BAO 2.0 ontology modularization framework**. The framework uses a layered architecture to abstract complexities from different sources. It provides modeling primitives of vocabularies, modules, axioms, and perspectives to develop heterogeneous ontologies.

**Figure 4:**
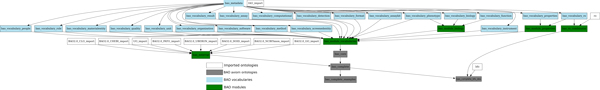
**BAO 2.0 ontology modularization framework implementation**. BAO 2.0 modularization framework provides effective software engineering methods to build complex ontologies. Shown are the current vocabularies, modules, and axiom files also indicating internal vs. external sources.

The modularization implementation is described in detail in Methods. Our modularization approach is illustrated in Figure 3. The modularization framework uses a layered architecture and uses the modeling primitives, vocabularies, modules and axioms. Vocabularies only contain terms (classes with subsumption only). Module layers enable combining vocabularies in flexible ways to create desired ontology structures or subsets. Axioms are separate files that do not contain any classes or properties. Classes and relationships are imported (directly or indirectly) from module and/or vocabulary files. The above mentioned classes in BAO 2.0 were created as separate vocabulary files. They were then imported into the bao_core file. BAO core only contains axioms incorporating BAO classes and BAO properties. In our modularization approach we separate external and internal sources. External modules (compare Figure 2) are generated as described in Methods. Overlap among external and internal classes and properties (i.e., those required in BAO core) are resolved using combinator modules, that is, external classes and properties are mapped (equivalence or subsumption) to corresponding BAO classes and properties. This approach assures that BAO core remains stable and independent from external sources that may change. The complete BAO includes external axioms and imports BAO core (indirectly importing all vocabularies and properties) and external modules (bao_complete file). Using this approach we also generated the BFO version of BAO. All internal and external vocabulary, module and axiom files are available via the BAO website (http://bioassayontology.org). Figure 4 shows the current implementation of the modularization illustrating vocabularies, intermediate modules, ontology axioms, BAO internal and external sources and their mappings.

### Modeling assays and results using BAO 2.0

In addition to the BAO modularized design and systematic construction, we also tried to make the definitions of concepts in BAO consistent. We especially defined bioassays with their essential components such as assay design method, endpoints, measure groups, and molecular participants. Figure 1 illustrates how assays are modeled by specifying information related to the biology (such as target and/or biological process), assay format, assay method (including assays design method and physical detection method, screened entity and endpoint (result) as described above. The BAO 2.0 architecture allows a more flexible definition of bioassays, for example the same biomolecule can participate in assays in different roles and functions. Important classes include:

• target: The target concept is defined by using the relationships has participant and has role. That is because targets are biological entities (i.e., participants) of assays that are playing the role target. Assays may have single or multiple targets depending on the assay type.

• biological process: A large number of assays are designed to measure outcomes of biological processes. Thus, based on the assay in study, we have written axioms for these information in the assay definitions.

• screened entity: This concept refers to a molecular entity with the role screened entity role.

• participants: Every assay has at least one participant, usually more. While axiomizing the assays, we try to define the particular roles that these participants play in the different assays. However, when we are not certain about the roles, we choose not to put axioms in order to avoid false reasoning cases.

• assay design method: Every assay has an assay design as the underlying method to generate a detectable signal and could correlate with the strength of the perturbation of the biological model system by the screened entity.

• physical detection method: An assay design method, generating a type of signal is linked to a corresponding detection method (the physical principle of detecting the signal), which is typically performed by a detection instrument.

The concepts listed above along with various other classes are used while modeling the concepts bioassay, measure group, and endpoint.

**Figure 5:**
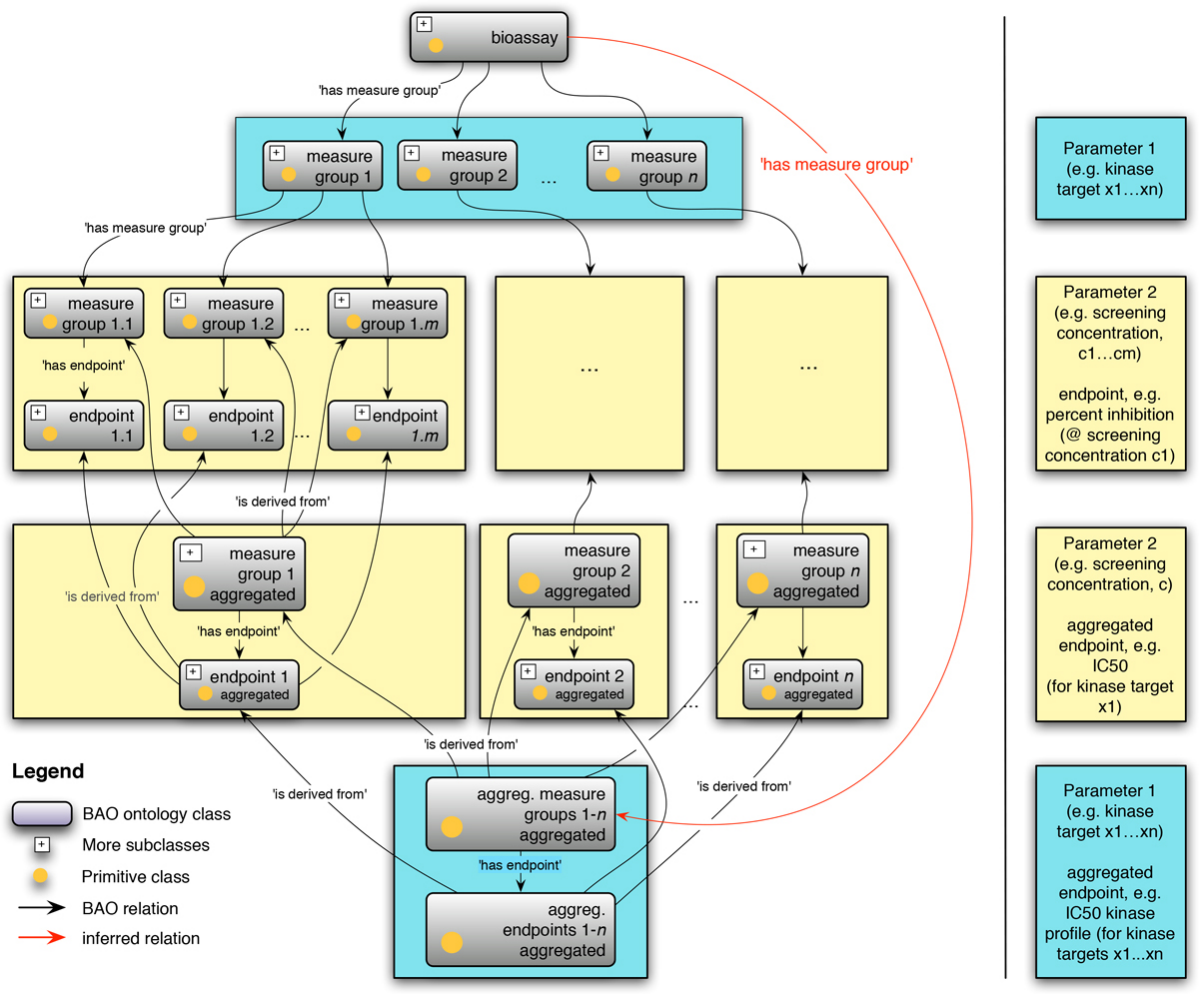
**Graphical illustration of BAO 2.0 measure group class definition**. The class measure group is used to group and link one or more sets of experimental results to one bioassay. By definition one assay can have multiple measure groups. The measure group contains overlapping axioms with the bioassay, which allows the reasoner to infer that the measure group is acting like an equivalent class of the bioassay; i.e., measure group is inferred as subclass of bioassay. Shown is an example of kinase concentration-response profiling panel assay, in which compounds are tested at m concentrations against n kinase targets.

We had previously introduced the concept measure group to link multiple endpoints to the same bioassay [28]. We have now generalized this model so that measure group can be derived from one or more measure groups. This allows the formal and iterative construction of more complex assays and endpoints that are derived from multiple measurements (Figure 5). The axiomatization was done in a way that infers measure group as a subclass of bioassay (compare Figure 1). The axiomatization was motivated by pragmatic considerations for the workflows and perspectives for organizing and analyzing the assay results, which is the core focus of BAO. It may be argued, that operationally it is not formally an assay; however that is not in conflict with the BAO perspective. It should be noted that BAO measure groups and results remain associated with their corresponding subclasses of bioassay, whose instances are procedurally, methodologically, and materially real. To understand better the relations between the concepts measure group and endpoint we explore them in more depth:

• The class measure group is a concept to group and link one or more (different) sets of experimental results to one bioassay. A bioassay can have multiple measure groups. A measure group contains overlapping axioms with the bioassay, which allows the reasoner to infer that the measure group is acting like an equivalent class of bioassay. This equivalence cannot simply be asserted. The measure group, in addition to holding the assay component metadata for each reported endpoint, also provides flexibility to generate different derived endpoints, e.g., IC50 (generated from several response values at different concentrations, i.e., concentration-response), or profile endpoints (e.g., a kinase panel assay). This can be formally done via derived measure groups, in cases where we have multiple measure group that vary in one parameter (such as concentration or kinase target).

• The class endpoint, alternatively called result, is a quantitive or qualitative representation of a perturbation (change from a defined reference state of the model system) that is measured by the bioassay. An endpoint consists of a series of data points, one for each perturbing agent employed by the assay. Every endpoint is obtained by using at least one measure group. For each endpoint, there exists a unit and a value, which is a number (e.g., float, which makes this concept a data property, and the concept is axiomized using a data property as opposed to an object property). For example, for a concentration endpoint (e.g., IC50), there exists a concentration unit and a concentration value, which is a float number (data property, not functional). Assays could have single or multiple endpoints depending on the assay type.

Endpoints are not used to handle the different measurements in the same assay. That is axiomized through the measure group concept. They may vary due to parameters such as time, concentration, target, and so on, or combinations. The formal definitions allow us to create individuals for different endpoints that might be using the same measure groups, i.e., results are measured once and different methods are applied on these measurements to find different derived endpoints. We can group different measure groups to define "intermediate" results. We can create profile endpoints and we can define profiles of intermediate aggregated measure groups (Figure 5). An endpoint individual is associated with a specific measure group and a specific compound combination and has a specific value and unit.

In BAO 2.0, endpoints are classified into several categories; the most important ones are concentration endpoint (which includes concentration response endpoint), response endpoint, protein substrate and ligand constant, and physical property endpoint. The class mode of action defines the functional effect and physical binding characteristics of the screened entity on the target using the subclasses ligand function mode of action (inhibition, activation, etc.) and ligand binding mode of action (reversible, irreversible, competitive, etc). Each endpoint is associated with a mode of action, e.g., IC50 and percent activation have inhibition and activation as the functional mode of action, respectively. The class signal direction defines how the functional effect of the perturbation corresponds to the intensity of the detected signal, i.e., increase or decrease with activation or inhibition. This is important to identify suitable counter screens; for example if the detected perturbation results in signal decrease in a cell-based assay, cytotoxic compounds may be detected as actives. The class endpoint action correlation defines if the endpoint value corresponds to increased or decreased functional effect (inhibition, activation). Both signal direction and endpoint action correlation are required to formally interpret the results, because the same perturbation (e.g., inhibition of substrate-protein binding by a competing ligand) may be measured via a different molecular entity with the role measured entity (e.g., substrate-bound protein or ligand-bound protein) and the effect can be expressed in different ways (e.g., normalized as remaining percent activity or percent inhibition). Further, depending on the assay design method, the same perturbation in the same model system may result in increased or decreased signal.

### Application to model LINCS profiling and panel assays and results

The concepts bioassay, measure group, and endpoint as described above enable the formal definition of panel and profiling assays such as those routinely run in the LINCS program. An effective modeling solution is relevant, because of the emphasis of LINCS to operate on result profiles and signatures, in contrast to individual endpoints. We define a panel assay as the parallel, spatially separate implementation of several identical assays, but that vary in one parameter (other than the screened entity), typically the target. A popular example is a kinase panel, for example the DisoveRx KINOMEscan assay that is also run at LINCS and in which compounds are screened against over 450 kinases in parallel. Similar to a panel assay, a profiling assay can generate a large number of readouts for any given tested compound, but all results are obtained from the same physical experiment, i.e., the same well. Such assays are also called multiplexed assays and rely on sophisticated assay methods and/or detection technologies that enable the detection of many signals in parallel, such as flow cytometry, mass spectrometry or imaging. One example also run at LINCS is the L1000 transcriptional profiling assay (*vide supra*). As illustrated in Figure 5, our approach would also allow to define concentration response (e.g., IC50) kinase profiling assays via iterative aggregation of sets of measure groups corresponding to two parameters, namely m screening concentration (values) and n kinase targets. The first aggregation by screening concentration (e.g., via curve fitting) defines the IC50 endpoint for each kinase and the second aggregation defines an IC50 kinase profile endpoint. An actual example of such a assay is the ActivX Biosciences KiNative assay, which is also run in the LINCS program. We have modeled several LINCS assays including KINOMEScan assay, transcriptional response profiling assay, cell cycle state assay. The specific instances of these assays including hundreds of kinase targets, transcribed genes, cell lines, etc was implemented in an application ontology and these assays and screening results are available in our LIFE software system [7].

An example of a phenotypic cell-based LINCS assay is the cell cycle state assay. It is also described in BAO 2.0. In the LINCS project, several small molecules that are known to function as kinase inhibitors were tested on cancer cell lines for their ability to arrest the mitotic cell cycle. This assay was modeled in BAO as follows: the assay design method is S phase assessment or M phase assessment method. The presence of the markers, namely, EdU and anti-MPM-2 antibody, indicates that cells have entered/completed S phase and M phase, respectively. Hoechst 33342 was used to stain nuclei from all cells to obtain the total cell count in the assay. The detection method is fluorescence microscopy and the measured entity is DNA. The assay readout parameters are intensity parameter and counting parameter. The intensity of EdU and MPM2 were measured in the nucleus and cytoplasm, respectively. The counts of Hoechst 33342, EdU and MPM2 positive cells were reported after the threshold to signal intensity of each marker was applied. The endpoint was derived from the assay readout parameters after normalizing with the assay controls. The endpoint for this assay is percent apoptotic cells, percent mitotic cells, percent interphase cells, percent DNA replicated cells, percent G2 arrested cells, and/or percent mitotic arrested cells. The cellular phenotype or its disposition is obtained by quantifying cells which are positive for each of these markers.

### Categorizing mechanistically related assays by inference

BAO 2.0 contains detailed description of a range of common HTS assay, including the categories: binding assay, cell cycle assay, cell viability assay, cytotoxicity assay, enzyme activity assay, gene expression assay, redistribution assay, and signal transduction assay. The essential information that was described for each assay type includes format, method (including assay design), detection, endpoint, and molecular and cellular entities and their roles, qualities and functions describing the biology of the system or which are key components involved in the assay design or detection methods. We have previously shown how promiscuous frequent hitter compounds (undesired assay artifacts) can be deconvoluted and categorized mechanistically based on detailed knowledge about the assays and their related design and detection methods [29]. However, using the previous version of BAO (1.6) these assays were not yet defined in a way that formalizes all necessary knowledge about their commonalities. This means that previously, in order to perform mechanism-based cross assay analysis, some human expert knowledge was required to identify and categorize related assays beyond their asserted annotations.

**Figure 6:**
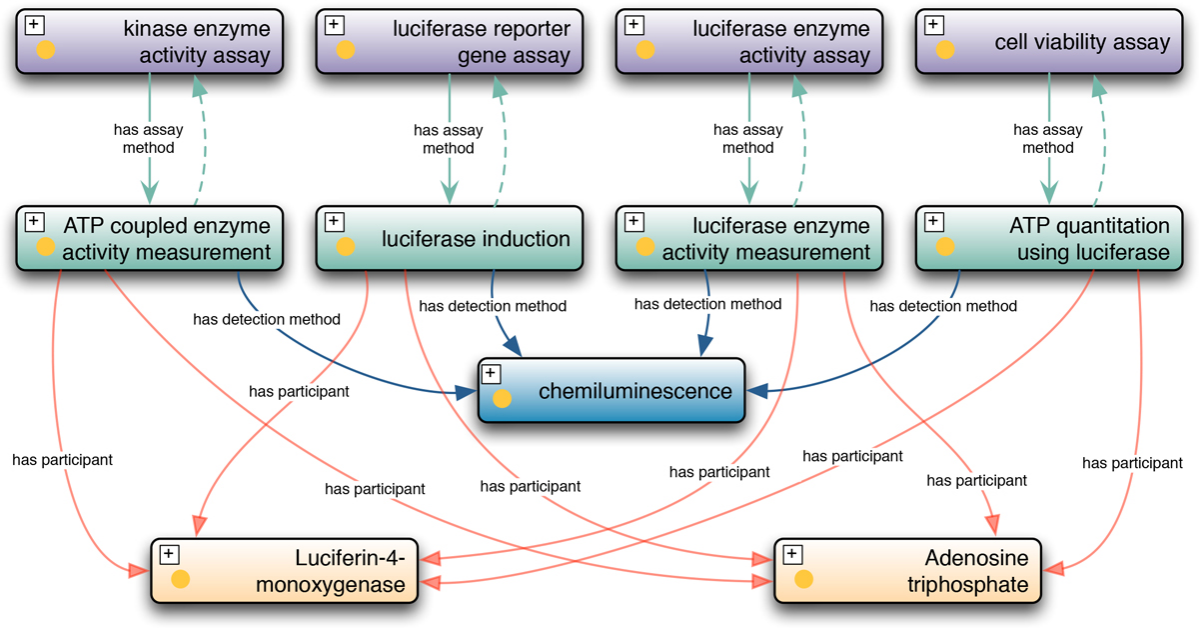
**Conceptual modeling of different luciferase assays**. Shown are bioassay, assay design method, physical detection method and participants (molecular entities with a specified role in the assay).

**Figure 7:**
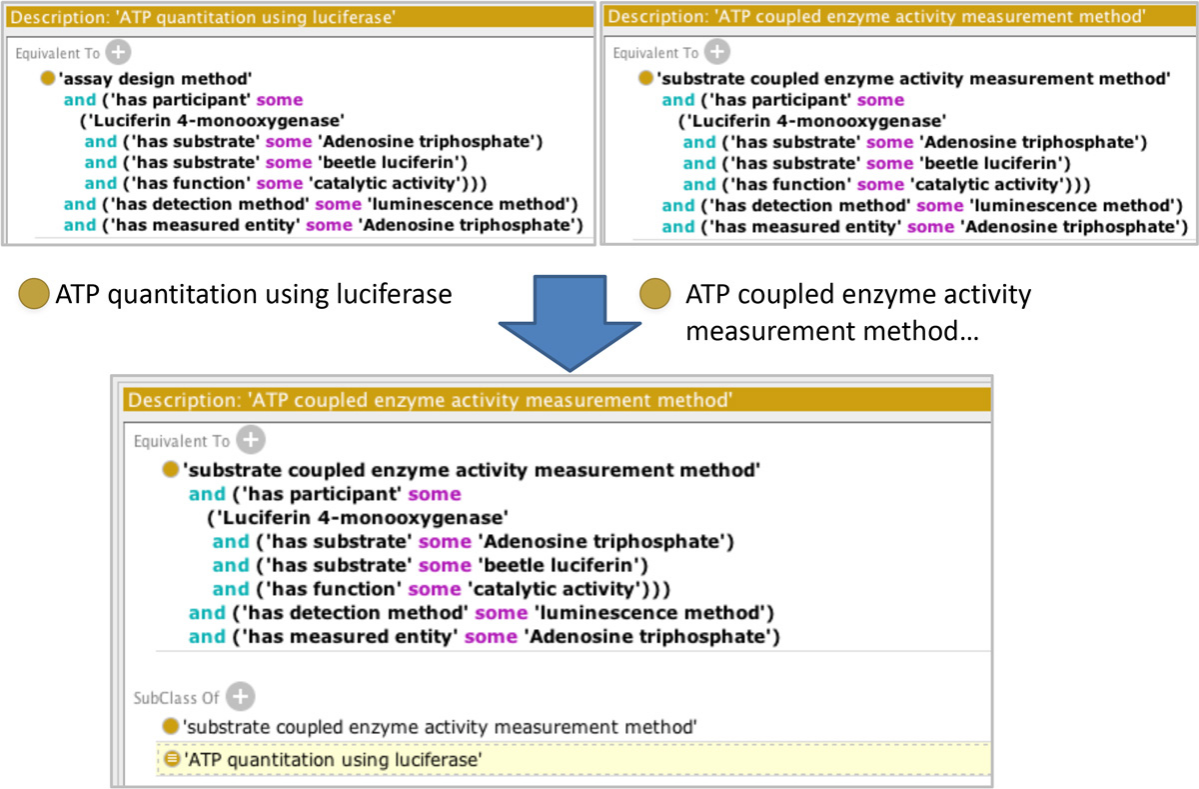
**Examples of luciferase assay design methods**. Shown are the asserted TBox of ATP quantitation using luciferase and ATP coupled enzyme activity measurement method and the inferred TBox in which the latter is classified as a subclass of the former.

**Figure 8:**
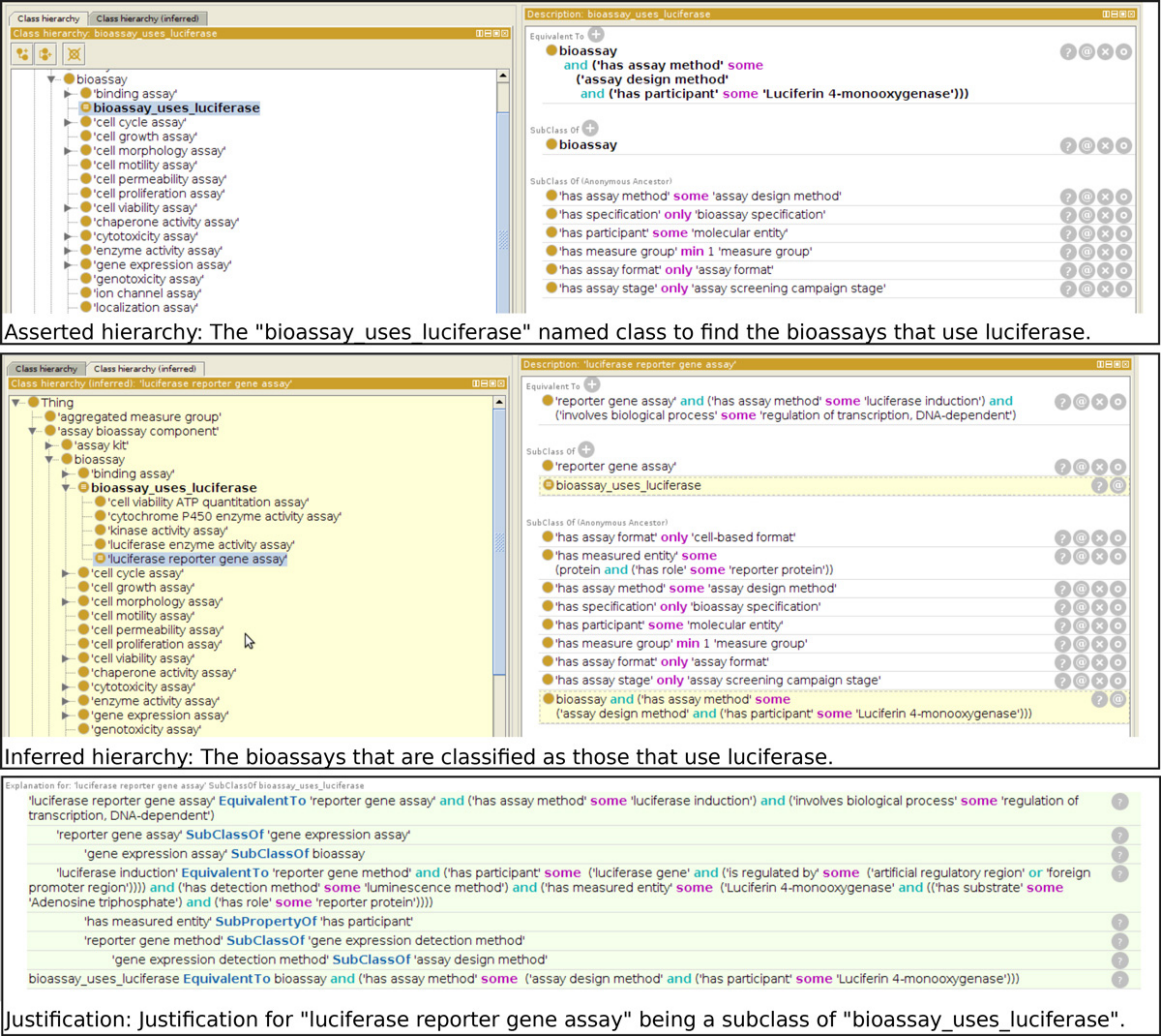
**An example that infers all bioassays in the ontology that use luciferase**. This example provides asserted and inferred hierarchies for bioassays that use luciferase as a participant. It also provides justification for luciferase reporter gene assay being a subclass of bioassay uses luciferase.

BAO 2.0 provides a framework that enables automated classification of assays into meaningful categories of interest, for example to aid in identifying common assay artifacts and their likely mechanism of action. We illustrate this using several related assays: luciferase reporter gene assay, cell viability ATP quantitation assay, cytochrome P450 enzyme activity assay, kinase activity assay, and luciferase enzyme activity assay. Of these, the reporter gene and cell viability assays are cell-based, while the others are biochemical assays. The modeling of these assays is illustrated in Figure 6. All assays use a different assay design method. Therefore they cannot be identified as mechanistically related based on that annotation alone. The physical detection method chemiluminescence is the same for all assays, but it is too generic to classify the assays by mechanisms that underlie artifacts, because luminescence can be generated by many methods. However, among these examples, all assays perform (in different ways) the luciferase-catalyzed chemical reaction of luciferin and ATP forming oxyluciferin and light (luminescence) and thus luciferase and ATP participate in all these assays, although in different roles. For example in the reporter gene assay the amount of expressed luciferase is quantified by the intensity of light (luminescence) produced in the presence of substrates, ATP, and luciferin. In the viability assays the proportion of living cells is quantified by measuring ATP content, again by the same reaction (with ATP as the limiting reagent in the role measured entity). Similarity ATP-coupled assays measure the residual amount of ATP (e.g., after a kinase reaction) by a coupled luciferase reaction. The P450 luciferin-coupled assay mentioned above measures the amount of luciferin generated after detoxification by cytochrome P450 enzyme activity. Luciferase enzyme activity assays quantify the biochemical luciferase enzyme activity by the intensity of light, again using the same chemical reaction. In BAO2.0 we modeled these assays with the necessary formalism to enable the reasoning engine to categorize the assays as mechanistically related. As an example, Figure 7 shows the asserted TBox of the assay design method ATP quantitation using luciferase and ATP coupled enzyme activity measurement method and the inferred TBox in which the latter is classified as a subclass of the former. For illustrative purposes we defined a class of all assays with an assay design method in which luciferase participates (in any role). The axioms and the asserted and inferred hierarchies are shown in Figure 8. All assays mentioned above are inferred as assays that use luciferase, thus illustrating how BAO formal assay definitions enable a classification based on the mechanistic principle of the assay (assay design method). This in turn classifies the assay based on likely common artifacts (e.g. compounds that stabilize or inhibit luciferase) [29]. Figure 8 also shows the justification for classifying the assays mentioned above under this category.

### Collaborative development and application of BAO to annotate assays

We had previously annotated (using BAO 1.6) a large set of assays from PubChem [28] and made these annotations searchable in BAOSearch [13], which is a Semantic Web application. These annotations were now mapped to BAO 2.0 and expanded to include additional information such as bioassay type, cell culture conditions, DNA construct details, roles, functions and qualities of molecular entities participating the the assays. 297 luciferase assays (*vide supra*) containing 328 measure groups are among the annotated assays, including the ones mentioned above and others. As explained above, the formalization of assays in BAO allows us to retrieve these assays based on a participant such as luciferase or ATP, even though these molecular entities were not explicitly annotated. A large project in which BAO has been applied and which in-turn significantly influenced the evolution of BAO is BARD [6]. In BARD, all MLP data, consisting of over 600 probe discoveries, are curated and annotated using controlled terms and organized into probe projects. BARD makes these data searchable in various ways and enables integrative analysis. During the development of BARD, data curation and annotation, the development of new terminology, and evolution of BAO has occurred in parallel. The development of terminologies and ontologies to annotate assays at Novartis also influenced our work in the BARD and BAO projects and highlights its relevance. We also applied BAO to define LINCS assays (*vide supra*); we make LINCS data searchable and explorable via the LIFE [7], which leverages Semantic Web technologies to integrate and search diverse data types. Our RegenBase project [30] also leverages BAO. BAO is also explored in the ChEMBL and PubChem projects, where BAO endpoints are used in RDF schema [16], and at PubChem. As another example from the pharmaceutical industry, a research group at Astra Zeneca is using BAO to annotate assays in the context of the Open PHACTS project (personal communications).

## Conclusions

We have developed BAO 2.0 as a reference for standard metadata terms and definitions required to describe relevant information of low and high-throughput drug and probe screening assays and results to enable effective data integration, aggregation, retrieval, and analyses. BAO 2.0 has been developed collaboratively to provide wider scope in describing and modeling diverse and complex assays. BAO is extended significantly with regard to the previous version using domain knowledge and data annotated in BARD and by other collaborators. We have described a flexible layered architecture to develop and integrate plethora of modules from established biomedical ontologies and upper level ontologies. Our modularization approach defines several integral distinct BAO 2.0 modules and separates internal from external modules and domain-level from structural components. This approach facilitates the generation/extraction of derived ontologies (or perspectives) that suit a particular use case or software application. We have generalized BAO to enable modeling of result profiles (signatures) generated in panel and profiling assays, for example those in the LINCS project. BAO leverages OWL DL (SROIQ(D)) to capture and formalize knowledge about assays and screening results and to enable computational systems to utilize knowledge. This enables the classification of assays and screening results into categories that relate to the assay model system, the biology (e.g., protein target or process), how a signal is generated and how it is detected, and screening results. We demonstrated inference reasoning capabilities of BAO to classify assays into categories that relate to how the assay works. This offers the potential to identify common promiscuous frequent hitters and their possible mechanism of action. We have leveraged BAO in software tools, such as the Semantic Web software applications BAOSearch, LIFE, and the BARD system. We continue to develop and expand BAO further with the goal to establish a standard to report chemical biology assays and their results. For example, to better describe the pharmacology of GPCRs, we recently developed a GPCR ontology framework [31]. We are also expanding BAO further to describe high-content phenotypic assays. BAO is currently used in several public and private screening projects and evaluated by a number of organizations and projects. The participation of groups from industry and academia to develop and use BAO illustrates the utility of the product as well as increasing public-private collaboration in pre-competitive areas, such as the development of standards and ontologies. BAO 2.0 is freely available from the BAO project website (http://bioassayontology.org) and the NCBO BioPortal. Additional file 1 contains BAO 2.0 ontologies as well as the examples illustrated in this manuscript.

## Methods

### BAO 2.0 development approach

BAO 2.0 was developed from BAO 1.6. It was performed in the following steps: First, upper level classes were created to include the various entities that participate in a bioassay, and their roles and qualities. Second, vocabulary files were created by moving the individual upper classes to the respective files. Third, all the vocabulary files were imported into a single file, called bao core (see Modularization below). Fourth, the upper level ontology, BFO was imported into bao complete and the various vocabulary files (either intact or separated as required) were moved to the respective upper level ontology classes. The process of importing external ontology modules, including object properties are described in detail below.

### Generating and processing external ontology modules

BAO is currently using excerpts from eleven external ontologies (including BFO): (1) Gene Ontology (GO); (2) Cell Line Ontology (CLO); (3) Unit Ontology (UO); (4) NCBI Taxonomy (NCBITaxon); (5) Human Disease Ontology (DOID); (6) Chemical Entities of Biological Interest (ChEBI); (7) UBERON (a comparative anatomy ontology); (8) Phenotypic Quality Ontology (PATO); (9) Information Artifact Ontology (IAO); (10) Relationship Ontology (RO); and (11) Basic Formal Ontology (BFO). The workflow for extracting external ontologies is as follows: Domain experts provide the list of concepts of interest and their ontology IDs. Based on these lists and the expression level of the external ontologies, we either use Java programs with OWL API to extract modules from the external ontologies of interest or we use the online tool OntoFox [32] to extract the concepts of interest. Several of the ontologies listed above are taxonomies, where we use OntoFox to avoid overlapping efforts and/or redundant code. Currently we use OntoFox for the ontologies listed below: (1) GO; (2) CLO; (3) NCBITaxon; (4) DOID; (5) ChEBI; (6) PATO; and (7) UBERON.

### BAO modularization implementation

The Web Ontology Language (OWL) [33] Description Logic (DL) - provides a rich set of constructors to model a domain of discourse. The DL expressivity comes with a substantial computational cost, as the state-of-the-art DL reasoners costs 2NExpcomplete (e.g., [22]). When the size of the ontology increases (number of axioms), the computational cost increases exponentially. In order to manage the size complexity, we provide a modularization methodology that preserves the required expressivity, yet being able to scale with the size of the ontology. Figure 3 shows the basic structure of the methodology.

The proposed modularization framework uses concepts from Directed Acyclic Graphs (DAG)s [34]. First, we determine the abstract horizon between TBox and ABox. TBox contains modules, which define the conceptualization without dependencies. These modules are self contained and well defined with respect to the domain of discourse. In these modules we provide concepts, relations, and individuals. The individuals are restricted to nominals, therefore they only act to close the class expressions. Figure 3 shows the main components of the framework: the top left boxes are physical files, i.e., each of them is an .owl file. They contain parts of our ontology, e.g., the top left file may contain everything of the domain of discourse that we think is necessary and important. We can have *n *of these modules.

Second, once the *n *modules are defined and if those modules have interdependent axioms, they are provided with another ontology (or module), which imports the necessary modules. At this level one could create any number of gluing modules, which import other modules without dependencies or with dependencies. At this level, the modules depends only on the modules of discourse. So, they all are combined in another physical .owl file, which we may call bao core (c.f., 3). The purpose of this core file is that it not only combines all of the submodules together (by referring to concepts from other physical files), it also is self-contained. This means that there is no outside term or relationship in this file.

Third, at this level we can design modules that import modules from our domain of discourse, and also from third party ontologies. Third party ontologies could be large, therefore a suitable module extraction method (e.g., OWL API) can be used to extract only part of those ontologies (*vide supra*). An example would be using a BFO term or a RO relationships. We would model this in the bao axiom level. We can have one bao axiom file or multiple files, each may be modeled for a different purpose, e.g., tailored for various research groups. Thus, bao Axiom 1 *. . . n *as seen in Figure 3. Once these ontologies are imported, the alignment takes place. The alignments are defined for concepts and relations using equivalence or subsumption DL constructs. The alignment depends on the domain experts' best guesses.

Forth, release the TBox based on the modules created from the third phase. Depending on the end-users, the modules are combined without loss of generality. With this methodology we make sure that we only send out physical files that contain our (and the absolute necessary) knowledge.

Fifth, at this level, the necessary modules ABoxes (again 1 *. . . n *ABoxes) are created. ABoxes can be loaded to a triple store or to a distributed file system (Hadoop DFS [35]) in a way that one could achieve pseudo-parallel reasoning.

Finally, using modules, we define *views on the knowledge base*. These are files that contain imports (both direct and indirect) from various TBoxes and ABoxes modules for the end-user. It can be seen as a *view*, using database terminology. In essence, we will be able to tailor these views based on the modules that we need. We expect that this methodology will speed-up the loading process, since only the necessary modules are loaded rather than every file that imports thousands of unnecessary and possibly redundant terms (e.g., due to potential loops in the imports). Therefore, BAO modules: (1) modify, expand, and maintain BAO independently; (2) use BAO in related efforts, such as knowledge reporting, more efficiently; (3) expand and synchronize BAO concepts in related efforts (e.g., BARD, LIFE, RegenBase, etc.); (4) reuse parts of BAO for different projects; (5) use other ontologies easily and without effecting BAO in order to support community efforts; and (6) provide transparent mapping of BAO to upper ontologies.

In summary, our methodology is as follows: (1) different files for different modules should be created and each module should contain all the concepts as a taxonomy file; (2) after the *n *modules are created as taxonomies, the core owl file should combine these modules; (3) the axioms related to the core ontology terms should be added to the core owl file; (4) once the core owl file is created that has nothing but the ontology's native concepts and axioms created by the native concepts, the third level file that has external ontologies should be created. The external ontologies can be added by using different combinations and related axioms can be added to the ontology at this level; (5) after creation of the one or more owl files that link different external ontologies and contain related axioms, individuals related with the ontology are added/loaded in the next level; (6) the view file that contains imports (both direct and indirect) from various TBoxes and ABoxes is created from user specifications. Based on this framework and methodology we have modeled the BAO 2.0. Figure 4 provides a complete description of the current vocabularies, modules, and axioms files and their connections developed for the ontology.

Our modularization framework differs significantly from existing methodologies: In decision making, a state represents a situation in which decisions should be made. An ontology provides a basic framework to represent situations in which decisions are made. Depending on the layer in which the decisions needs to be made, a state can represent from low-level signals to high-level mental abstractions. Therefore, state abstraction provides the basis in which layer-wise decisions are made. OWL ontologies provides mechanisms such as "owl:import" to represent state abstractions. But this has not been explicitly studied in large scale ontologies. Our modularization framework assesses the capabilities of OWL ontologies to represent state abstractions in different complexities.

Ontology interpretation provides mechanism to represent vocabularies for classes, roles, and individuals. Without any other assumption, the interpretations of these entities provides the state of the system. These entities are analogues to low-level sensations from perceptions. Our modularization framework captures these representations in the vocabulary layer. One can use these representations for tasks such as to populate drop-down menus in a web-application etc. These representations are at its basic levels and the system does not assume any constraints. Having provided constraints leads to OWL ontologies to represent state abstractions.

In order to provide additional information related to basic entities, the next step is to enrich the state with constraints. In first-order-predicate logic, constraints are provided by axioms. Therefore, in OWL ontologies, we use axioms as the method to provide the constraints, hence, the state abstractions. The "modules" in the modularization framework provides different constraints. The modules are connected though the "owl:imports" mechanism, and the constraints are provided by OWL constructs available in SROIQ(D) description logic. Therefore, at each layer, do-main experts provide axioms for the best of their knowledge. The modularization framework provides hard boundaries in which, a domain user can extract constraints. This partially addresses one of the problems in ontologies: axioms extraction, which has NP-hard complexity. The hard boundaries provides decision points where abstract state of another system should have been extracted, for example, one can extract whole BAO abstract state without reference to a upper-level ontology such as BFO.

The proposed modularization framework provides basic steps to implement our knowledge base reporting (KBR) application. It needs to infer knowledge from massive ABoxes with parallel reasoning using frameworks such as Map-Reduce. KBR needs decision layers in which to knowledge to be reported, and our modularization framework provides those decision points.

In artificial intelligence, state representations and state abstractions are an open problem. OWL ontologies, with respect to first-order-predicate logic, provides methods to represent knowledge, but, to our knowledge the state abstraction is not discussed widely. Our modularization framework addresses these problems and possibilities in which state abstraction can be generalized.

### Assay annotations: terminology alignment, reformatting and processing

As described in our earlier publication [12], assays from PubChem were annotated using BAO 1.6 terminology. These existing annotations were mapped to corresponding BAO 2.0 classes and annotations were expanded including cell culture conditions, DNA construct, quality, role and function of molecular entities. In addition, the object properties and data properties were refined; many were imported from the RO. Cell line, gene and protein names were standardized by importing the nomenclature from CLO or specific repository, NCBI or HUGO, and UniProt, respectively. In total, 1,000 assays in the PubChem database were annotated using BAO 2.0. These are leveraged in BARD; however, BARD includes all assays and results generated by the MLP screening centers (*>*6, 000) and organizes them by probe projects (*>*600). In the process of annotating assays, new terms were collected and subsequently mapped or added to BAO manually (after expert review). In addition, we incorporated terms from some of the Novartis ontology modules and terms requested by other collaborating group (e.g., Astra Zeneca). Assay annotations were captured in a spread sheet with column headers that correspond to BAO classes or relations. For the luciferase assays, we translated the columns headers for the most important annotations and their contents into triples (by mapping column headers to corresponding relations) and loaded them into a RDF triple store as previously described. Figure 8 shows an example where we have used BAO 2.0 to infer all bioassays that use a method in which Luciferin 4-monooxygenase is a participant. We have defined the equivalent class bioassay uses luciferase as bioassay ⊓ ∃'has assay method' ('assay design method' n ∃'has participant' 'Luciferin 4-monooxygenase'). OWL DL reasoners infer that cell viability ATP quantitation assay, cytochrome P450 enzyme activity assay, kinase activity assay, luciferase enzyme activity assay, and luciferase reporter gene assay are indeed luciferase assays. Figure 8 provides justification for luciferase reporter gene assay being a subclass of bioassay uses luciferase. This allows us to identify assays that are annotated with any of these assay design methods as assays that use luciferase, which is relevant to identify assay artifacts across various different bioassays.

## Competing interests

The authors declare that they have no competing interests. The views presented in this paper do not necessarily represent or reflect those of the funding organizations.

## Authors' contributions

SA and UDV contributed equally to the presented work. UDV, SA, AM, KS, PAC, JAB, AS, AJM, MR, SB, VL, and SCS developed the ontology (terminology, definitions, properties). UDV mapped BAO concepts to BFO. SA, HK, UV, and SCS developed the modularization method and OWL DL implementation. SA and HK developed the ontology supporting tools. HK, UDV, AJM, PAC, SB, SA, and SCS provided the ontology alignment with external ontologies and BARD. SA, UDV, HK, AK, DT, and SB developed ontology applications (assay annotation, software implementation). CC, VL, and SCS conducted ontology releases, QC, support, and CC maintains the BAO website. SCS envisioned and designed the BAO project. SA and SCS wrote the paper with contributions from UDV, HK, UV, and VL.

## Supplementary Material

Additional file 1 **BAO 2.x build 2866**. BAO version 2.x (build 2866) is a collection of OWL files that describe our domain of discourse. These files are serialized in OWL XML file format. In order to view and perform reasoning of BAO, we recommend of using a standard OWL editor that support *SROIQ*(*D*) DL constructs, such as, Protégé-4.3 open source ontology editor and knowledgebase framework (http://protege.stanford.edu). In addition, up-to-date BAO releases are freely available from the BioAssay Ontology (BAO) project website (http://bioassayontology.org/wp/bao) and the NCBO BioPortal. Click here for file

## Declarations

The publication costs for this article were funded by the Center of Computational Science of the University of Miami.

This article has been published as part of *Journal of Biomedical Semantics *Volume 5 Supplement 1, 2014: Proceedings of the Bio-Ontologies Special Interest Group 2013. The full contents of the supplement are available online at http://www.jbiomedsem.com/supplements/5/S1.
